# Case-based pharmacotherapeutic learning structured around the prescribing optimization method and the WHO 6-step model: Medical students’ views from an exploratory questionnaire study

**DOI:** 10.1007/s00228-026-04184-7

**Published:** 2026-09-19

**Authors:** Massimiliano Carifi, Fabio Furchì, Naldy Parodi López, Joost D. Piët, Jelle Tichelaar, Fabrizio De Ponti, Susanna M. Wallerstedt

**Affiliations:** 1 Pharmacology Unit, Department of Medical and Surgical Sciences, Alma Mater Studiorum, University of Bologna, Bologna, Italy; 2 Department of Pharmacology, Sahlgrenska Academy, University of Gothenburg, Box 431, Gothenburg, SE-405 30 Sweden; 3 Unit of Clinical Pharmacology, Department of Pharmaceuticals, Sahlgrenska University Hospital, Gothenburg, Sweden; 4 Department of Internal Medicine, Unit Pharmacotherapy, Amsterdam UMC, Vrije Universiteit, De Boelelaan 1117, Amsterdam, 1081 HV The Netherlands; 5 Research and Expertise Centre in Pharmacotherapy Education (RECIPE), De Boelelaan 1117, Amsterdam, 1081 HV The Netherlands; 6 Interprofessional Collaboration and Medication Safety, Faculty of Health, Sports and Social Work, InHolland University of Applied Sciences, Amsterdam, The Netherlands; 7 Centre for Health Technology Assessment, Sahlgrenska University Hospital, Region Västra Götaland, Gothenburg, Sweden

**Keywords:** Education, Medical programme, Pharmacotherapy, Polypharmacy, Prescribing, Teaching

## Abstract

**Purpose:**

Pharmacotherapy is a core competence for physicians and case-based learning is a valuable teaching method for developing this competence. This study explored medical students’ views on structuring pharmacotherapeutic education around either the Prescribing Optimization Method (POM), starting from the medication list, or the World Health Organization (WHO) 6-Step Model, starting from the patient’s presentation.

**Methods:**

Students who had experienced both the POM and the WHO 6-Step Model, featuring cases involving multiple medications, during an elective pharmacology clerkship at the University of Bologna, were invited to complete an anonymous questionnaire. Participants rated their agreement with a statement regarding the usefulness of each structure for developing pharmacotherapeutic knowledge (1 = strongly disagree, 5 = strongly agree), indicated their preferred structure, and provided comments explaining their choice and contexts in which each structure was considered useful.

**Results:**

All students participating in the clerkship shared their views (*n* = 27). Their ratings of each structure’s usefulness for developing pharmacotherapeutic knowledge were 4.3 ± 0.8 (POM) and 4.1 ± 1.1 (WHO). Thirteen students preferred the POM, often referring to its comprehensive medication focus in the context of polypharmacy, and the value of such guidance in the undergraduate learning process. Twelve students preferred the WHO 6-Step Model, often referring to its alignment with clinical practice and the learning it offers for their future professional role.

**Conclusion:**

Students perceived both structures as useful for pharmacotherapeutic learning; future studies should examine whether these approaches improve prescribing competence or therapeutic reasoning performance.

## Introduction

As pharmacotherapy is a fundamental competence for physicians, all medical graduates must master the basics. This encompasses not only a solid foundation in knowledge of diseases, pharmacology, and patient consultation, but also the ability to apply this knowledge in clinical decision-making. Indeed, pharmacotherapy is a complex task, requiring differential diagnosis as well as therapeutic reasoning, often demanding metacognition [1]. It can also can be considered to represent the highest level of cognitive skills―at the top of Bloom’s taxonomy [2]―as it involves creating new decisions based on individual circumstances. The importance of effective pharmacotherapy education cannot be overstated; although the quantification of harms related to medications from a healthcare perspective is challenging [3], medication-related hospitalisations and deaths that might have been prevented do occur [4, 5].

Case-based learning has emerged as a valued pedagogical approach, promoting teamwork and communication as well as critical thinking [6]. Two prominent frameworks for structuring case-based pharmacotherapy education are the Prescribing Optimization Method (POM) [7] and the World Health Organization (WHO) 6-Step Model [8]. The POM, specifically focusing on polypharmacy, emphasizes a systematic approach starting from the medication list and aiming to optimise pharmacotherapy by identifying potentially inappropriate medications, omissions, and drug-related problems. In this context, the Screening Tool of Older Person´s Prescriptions (STOPP) and the Screening Tool to Alert doctors to Right Treatment (START) can be useful, originally intended as an easy and time-efficient tool for the busy prescriber in day-to-day practice [9], and since then updated and expanded in two steps [10, 11]. In contrast, the WHO 6-Step Model begins with the patient’s clinical presentation and provides a structured process from differential diagnosis to appropriate treatment selection and the planning of monitoring and follow-up [8].

Within the European Clinical Pharmacology and Therapeutics Teach-the-Teacher (CP4T) project, the POM was the main educational structure used in international teaching sessions [12]. The WHO 6-Step Model, an update of which was also planned within the project [13], was applied on one occasion and was well received based on spontaneous feedback from participating teachers. This study contributes to clinical pharmacology education by exploring medical students’ perceptions of two differently structured approaches to therapeutic reasoning: one that begins with the medication list and one that begins with the patient’s clinical presentation, both addressing similar learning objectives and being applicable to patients with multiple medications.

## Methods

### Study design and setting

This questionnaire study was conducted during an elective pharmacology clerkship in the medical programme at the University of Bologna, Italy, primarily intended for fourth-, fifth-, and sixth-year students of the programme taught in Italian, and the international equivalent taught in English. It was approved by the Bioethics Committee (protocol no. 0252807). The students can choose to attend the program over two to six weeks (6 weeks correspond to 6 European Credit Transfer System (ECTS) credits) during the first semester of each year. The clerkship during which this study was performed consisted of three or four weekly sessions held between 23 September and 30 October 2025. The targeted students had previously received teaching in basic and clinical pharmacology where they are introduced to the WHO 6-Step Model, but they had not received training in either the POM or the WHO 6-Step Model before the clerkship.

### Educational intervention

**Fig. 1:**
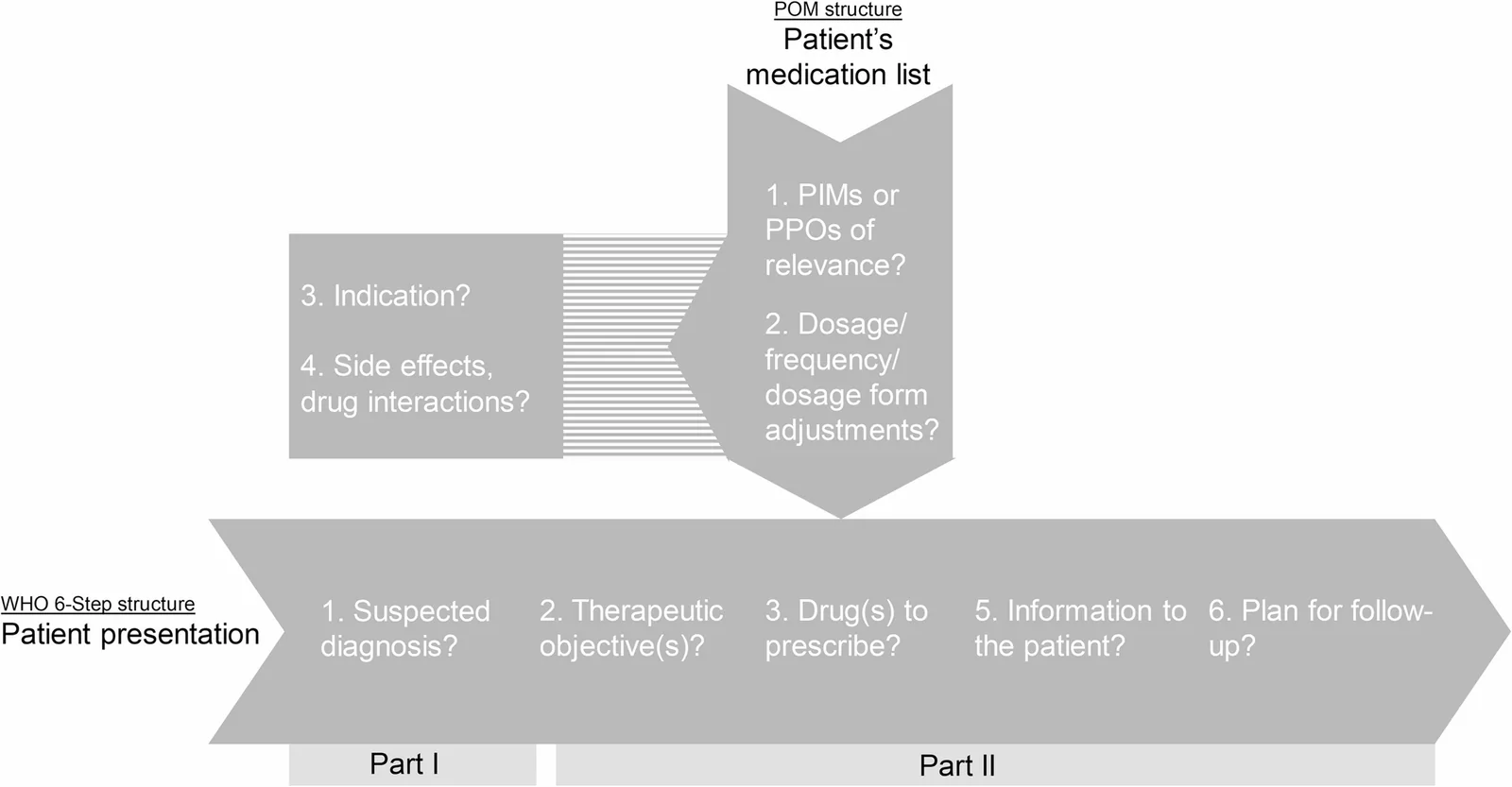
A schematic overview of the educational sessions performed in this study, structured around to the POM and the WHO 6-Step Model, with the latter divided into two parts. In the former, the original question regarding the patient’s adherence to their medication schedule was replaced with an instruction for students to determine the indication for each medication, to better align with a theoretical education session. In the latter, the fourth step—conveying adequate information to the dispenser or administrator to enable practical implementation of drug treatment—was omitted in the educational session for a similar reason

During the clerkship, the medical students participated in case-based pharmacotherapy teaching sessions that were primarily structured around the POM; the WHO 6-Step Model was tested once. The two structures were used with minor modifications (Fig. 1). In the POM, the original question regarding the patient’s adherence to their medication schedule was replaced with an instruction for students to determine the indication for each medication, to better align with a theoretical teaching session. In the WHO 6-Step Model, the fourth step—conveying adequate information to the dispenser or administrator to enable practical implementation of drug treatment—was omitted for a similar reason. Furthermore, the third WHO step was adapted from the original P(ersonal)-drug concept to better align with contemporary clinical practice, in which recommendations and clinical guidelines play a more prominent role. All educational sessions lasted approximately two hours, were built on team-based learning [14] with about five students in each team, and followed a repeatable model designed to integrate theoretical knowledge with practical applications. All cases were based on common clinical scenarios involving multiple medications and were of moderate complexity, making them suitable for medical students in their second half of the curriculum. Standardised teaching materials were used, including predefined clinical cases, standardised instructions, and common teaching resources. Two teachers (MC, FdP) and a senior student (FF) facilitated the sessions and were involved in both types of sessions in order to limit variability between the two approaches.

Each POM-structured session opened with an introductory briefing of that session’s clinical case, recalling key pathophysiological and pharmacotherapeutic aspects, and with reference to current guidelines. This was followed by the presentation of the case’s medication list representing polypharmacy (more than five medications, up to eleven), which served as the basis for the team discussions including the patient’s clinical history and current therapy with the aim of proposing any necessary changes, such as dosage adjustments or drug discontinuations. The students were instructed to apply the third version of the STOPP/START criteria [11] to screen for potentially inappropriate medications or potential prescribing omissions.

The WHO 6-Step-structured session opened, after an introduction to the framework, with the session’s clinical case itself, including the patient presentation and differential diagnosis. Once the diagnosis was disclosed halfway through the session, the discussion continued with pharmacotherapeutic management, taking relevant recommendations and guidelines into account. The clinical case concerned an older woman presenting with typical symptoms of polymyalgia rheumatica and sleeping problems, where diagnostic laboratory tests were to be considered. Thereafter, appropriate drug treatment was to be determined. In this process, the students were expected to consider both medications for symptom relief and preventive treatment aimed at reducing anticipated adverse effects, including those related to potential drug interactions. The case started with four medications and ended with a total of seven medications in the medication list.

At the end of both POM- and WHO 6-Step-structured sessions, a collective debriefing was held to discuss the proposed decisions, to share the reasoning that emerged within the teams, and to summarise the main learning points. This phase represented a moment of critical reflection and peer-to-peer discussion, helping to value different perspectives and recognise the limits of individual reasoning.

### Participants and data collection

Students taking part in the educational session on 9 October 2025 (*n* = 27) that was structured according to the WHO 6-step model were invited to participate in the present study. All these students had previously participated in nine educational sessions structured according to the POM. The students’ participation in this study did not influence grading or assessment.

Data collection was conducted after the educational session and using the anonymous voting system Mentimeter. The students were asked to rate their agreement with a statement on the usefulness of each method for developing pharmacotherapeutic knowledge, on a 5-point Likert scale from 1 = strongly disagree to 5 = strongly agree. They were also asked to indicate their preferred structure and provide comments explaining their choice. Furthermore, they were invited to describe various contexts in which they considered a structure according to the POM and the WHO’s 6-Step Model most useful.

### Data analysis

Descriptive analyses were performed using SPSS Statistics for Windows, version 27.0 (IBM Corp., Armonk, NY, USA). Free-text responses were thematically analysed, using an inductive approach guided by the questions “What are the reasons for preferring each method?” and “In what context is the respective structure considered useful?”, with new reasons/contexts noted as they emerged from the text. This analysis was conducted independently by three authors (MC, FF, SMW), followed by a consensus discussion in which co-authors were involved and disagreements resolved.

## Results

All 27 medical students participating in the educational session voluntarily participated in the study (14 men, 11 women, two did not provide information regarding gender; 20 were 20‒24 years; 16 were in the final year of the medical programme, two in the fifth year, five in the third year, two before their third year, and two did not provide this information). The students rated both structures highly in terms of usefulness (Table 1). Thirteen students (48%) preferred the POM structure, twelve (44%) preferred the WHO 6-Step structure, and two (7%) expressed no preference.

**Table 1:** Medical students’ views on case-based pharmacotherapeutic education structured according to the POM or the WHO’s 6-Step Model. Values are presented as median (IQR), mean ± SD, or number of students, respectively

		POM	WHO 6-Step Model
Agreement with the statement…^1^	“I find the [POM or WHO 6-Step Model] helpful for developing the pharmacotherapeutic knowledge required in my future work as a physician”	4 (4‒5) 4.3 ± 0.8	4 (3‒5) 4.1 ± 1.1
Preference^2^		13	12
Reasons favouring the structure^3^	More closely aligned with clinical practice	0	12
	Provides a framework that reduces the risk of missing recommendations	11	0
	Clear guidance early in the learning process	7	0
	Clear guidance later in the learning process	0	2
	Provides knowledge about medications	2	0
	Facilitates interaction with teachers/between students	0	4
	Facilitates in-depth reflections	2	0
Context where the respective structure was considered useful^3^	Clinical work, including medication review	8^4^	12^5^
	Polypharmacy and complex cases	7	1
	Straight-forward cases	0	3
	For team discussions	2	3
	For learning drug treatment/common medications	2	4
	When time allows	1	0

*IQR* interquartile range, *POM* Prescribing Optimization Method, *SD* standard deviation, *WHO* World Health Organization

^1^On a scale from 1 = strongly disagree to 5 = strongly agree

^2^Two students had no opinion

^3^Interpreted from free-text answers regarding why the selected structure was preferred

^4^3 and 1 students mentioned the general practice setting and the hospital setting, respectively

^5^4 and 3 students mentioned the general practice setting and the hospital setting, respectively

Students who preferred the POM primarily described its systematic structure and comprehensive medication focus as key reasons, offering clear guidance in the undergraduate learning process (Table 1). Quotes that illustrate this view are: “With the training we currently have, we are more lacking in the pharmacological aspect; therefore, the POM enables us to work better on the specific gaps in our type of training” and “POM allows gaining a higher confidence in handling and managing drugs as a beginner”. Conversely, students who preferred the WHO 6-Step Model emphasised its alignment with clinical practice, with some highlighting its usefulness in providing guidance in the professional learning process. Quotes that illustrate this view are: “I feel that it comes closer to the actual clinical reasoning that will be required of me once I have graduated” and “I think it’s more useful for my future clinical practice”. Another quote illustrates the value of both structures: “I think the POM might be more useful as a first time approach, to gain some confidence and learn a systematic method. After that, maybe the 6-step method is nearer to the clinical practice”.

Both structures were considered useful in clinical work including medication reviews. For the POM structure, the context of polypharmacy emerged in quotes like: “I consider it more helpful in patients with polypharmacy because it allows me to see every drug together with its benefits and side effects”. The usefulness of the WHO 6-Step structure for clinical work is illustrated in the quote: “In a prescription and approach to a patient, with an organic plan on how to treat considering more aspects of the disease and life of the patient”.

## Discussion

Our findings show that both the POM and the WHO 6-Step Model are valued by medical students in case-based pharmacotherapy education focused on patients with health conditions justifying the prescribing of multiple medications. The former was considered to provide a medication-focused framework for learning during undergraduate education. The latter was considered to be more closely aligned with clinical practice, providing a framework for professional learning.

The preference for the POM structure among half of the students may reflect an awareness that they need to acquire broad and specific knowledge related to medications. In this context, the POM structure, which focuses on the medication list and specifically addresses patients with polypharmacy [7], may be valuable. A similar approach is used in the later-developed Systematic Tool to Reduce Inappropriate Prescribing (STRIP), which also starts from the medication list and has likewise been reported to be appreciated by medical students [15]. By representing what is generally considered inappropriate and appropriate drug treatment in older people, the use of the STOPP/START tool may contribute to the first level of Bloom’s taxonomy [2]—factual knowledge—and serve as a foundation for reaching higher cognitive levels. The fact that several students highlighted that the POM offers clear guidance for acquiring medication-related knowledge during the undergraduate learning process supports this reasoning. The perceived value of a theoretical knowledge base is also supported by findings from a qualitative analysis of aspects that medical students consider important during the medical programme for developing basic prescribing skills, in which this theme emerged [16]. Furthermore, the student comment that the POM is useful when time allows may suggest that it has particular value in the educational context, where time constraints are generally less pronounced than in clinical practice. Indeed, although the average time for screening using the original STOPP/START tool reportedly was 1.5 min [9], the updated expanded versions are substantially more time-consuming, and their use in unfamiliar cases by an inexperienced assessor is likely to take considerably more time. Furthermore, the identification of such criteria using clinical data may not be as simple as it may appear from ready-made cases [17]. Indeed, reasonable uncertainties surrounding diagnoses and drug treatment add additional challenges.

Conversely, students who favoured the WHO 6-Step structure highlighted its alignment with clinical practice, which, for the physician, starts with the patient’s presentation, and where the diagnostic and therapeutic processes are closely connected. Indeed, the POM is primarily intended to optimise existing polypharmacy and can therefore be regarded as a reactive approach, whereas the WHO 6-Step Model is proactive, aiming to ensure appropriate prescribing from the outset. Comments by some students indicating that the WHO model as an educational structure offers advantages when pharmacotherapy needs to be integrated into the broader clinical context for decision-making, suggest that this structure can represent higher cognitive levels of Bloom’s taxonomy, at least when the case involves multiple differential diagnosis and pharmacotherapeutic considerations. It should be noted that tools such as the STOPP/START criteria can also be integrated into the WHO 6-Step Model workflow, to help identify medications generally considered inappropriate or appropriate in the third step. Indeed, although identified STOPP/START criteria only rarely merit action when individual circumstances are considered [18], the integration of practically useful medication knowledge into the medical curriculum has been recognised as a valuable educational strategy for supporting medical students’ learning and development of pharmacotherapeutic competence [19]. Against this background, it should also be noted that the current abundance of guidelines and guidance tools [20], as well as the availability of clinical decision support systems [13], are not mentioned in the 30-year-old WHO 6-Step Model. Furthermore, shared decision-making is not referred to in the WHO 6-Step Model [13], although it could be considered part of the third step.

Interestingly, the students’ preferences for either structure were nearly evenly split. In addition to indicating diversity in cognitive levels and learning preferences, the finding that both structures were highly valued may reflect the usefulness of case-based learning as a pedagogical format. This interpretation is consistent with a recent qualitative study exploring factors that medical students consider important for enabling them to treat patients with medications, in which case-based teaching emerged as a valued learning format [19]. Furthermore, one could speculate that any teaching intervention might be perceived as beneficial, given that few final-year medical students feel confident in their prescribing skills [21]. Rather than promoting one method exclusively, educators might thus consider integrating both approaches to facilitate learning at various cognitive levels. The POM may be particularly useful in undergraduate education when teaching has progressed to more complex patients with multiple medications. The WHO 6-Step Model, which mirrors professional practice, allows complexity to increase throughout the learning process, from straightforward cases early on to more complex cases later.

From an examiner’s perspective, given that medication-related examination questions in clinical courses tend to focus on drug selection and factual recall [22], case-based sessions structured around the WHO 6-Step Model may offer a valuable opportunity to address other components of the prescribing process, including pharmacotherapy-related differential diagnosis, goal setting, patient counselling, and follow-up monitoring. Nevertheless, the fourth step, prescribing appropriately within an electronic health record system, is likely best acquired and examined through workplace-based training, where students can make use of integrated clinical decision support systems and gain experience in identifying information relevant to individual patient care.

An important limitation of the present study is that the students were exposed to the POM multiple times, including various case scenarios, and the WHO 6-Step Model only once. The fact that different cases were used in the sessions is also an obvious limitation. Nevertheless, both types of teaching sessions involved patients treated with multiple medications. In the context of polypharmacy, it should be noted that the definitions vary [23] and that the number of medications in a medication list may rather reflect the burden of disease [24], which in turn may indicate the complexity of drug treatment. Moreover, the number of medications is not a valid indicator of prescribing quality [25]. Other limitations include the small sample size and single-institution setting. The elective nature of the clerkship may further limit the generalisability of the results, as participants may have had a pre-existing interest in pharmacology. Furthermore, both structures were used in the context of team-based learning, and students’ views may be different in an individual learning context. It may also be considered a limitation that no method-specific questions regarding the utility of either teaching approach were included. However, the exploratory analysis of the free-text responses provided unbiased insights into the students’ views. Finally, it must be noted that the current study did not evaluate the outcome of the two educational structures; further research regarding educational structures and acquired prescribing skills would add important information.

In conclusion, this exploratory single-centre questionnaire study indicates that medical students perceive both POM- and WHO 6-Step Model-structured sessions as useful and complementary approaches to case-based pharmacotherapeutic learning about patients with multiple medications. POM is primarily valued for its comprehensive medication focus and support in addressing polypharmacy, whereas the WHO 6-Step Model is valued for its alignment with clinical practice and preparation for the physician’s role. Further research should evaluate whether integrating these approaches improves objectively assessed prescribing competence.

## Data Availability

The datasets generated and analysed during the current study are available upon reasonable request to the corresponding author. Teaching materials, including cases and instructions, are available upon request.
